# *N*-(β-Carb­oxy­eth­yl)-α-isoleucine

**DOI:** 10.1107/S160053681205146X

**Published:** 2013-01-04

**Authors:** Irene Nehls, Olaf Hanebeck, Roland Becker, Franziska Emmerling

**Affiliations:** a Federal Institute for Materials Research and Testing (BAM), Richard-Willstaetter-Strasse 11, D-12489 Berlin, Germany; b SGS Institut Fresenius GmbH, Tegeler Weg 33, D-10589 Berlin, Germany

## Abstract

The title compound, {2-[(2-carbamoyleth­yl)amino]-3-methyl­penta­noic acid}, C_9_H_18_N_2_O_3_, is of inter­est with respect to its biological activity. It was formed during an addition reaction between acryl­amide and the amino acid isoleucine. The crystal structure is a three-dimensional network built up by inter­molecular N—H⋯O and O—H⋯N hydrogen bonds.

## Related literature

For toxicological investigations on acryl­amide, see: Besaratinia & Pfeifer (2007 ▶); Parzefall (2008 ▶); Bowyer *et al.* (2009 ▶); Wang *et al.* (2010 ▶); Mei *et al.* (2010 ▶); Koyama *et al.* (2011 ▶); Lee *et al.* (2012 ▶); Nixon *et al.* (2012 ▶); Rice (2005 ▶). For directives on monitoring acryl­amide in drinking water, see: EU (2000 ▶). For the determination of acryl­amide in different media, see: Zangrando *et al.* (2012 ▶); Marin *et al.* (2006 ▶); Lucentini *et al.* (2009 ▶); Keramat *et al.* (2011 ▶); Tareke *et al.* (2002 ▶); Pittet *et al.* (2004 ▶); Castle & Eriksson (2005 ▶); Mizukami *et al.* (2006 ▶); Dias Soares *et al.* (2009 ▶); Alpmann & Morlock (2008 ▶); Preston *et al.* (2009 ▶); Perez & Osterman-Golkar (2003 ▶).
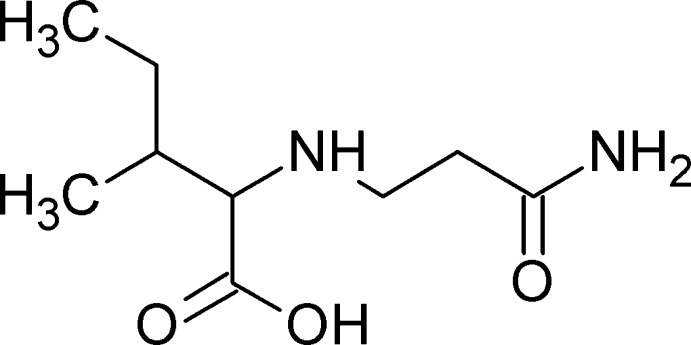


## Experimental

### 

#### Crystal data

C_9_H_18_N_2_O_3_
*M* *_r_* = 202.25
Orthorhombic, 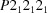

*a* = 5.2989 (17) Å
*b* = 9.024 (3) Å
*c* = 23.268 (7) Å
*V* = 1112.6 (6) Å^3^
*Z* = 4
Mo *K*α radiation
μ = 0.09 mm^−1^
*T* = 298 K
0.64 × 0.06 × 0.06 mm

#### Data collection

Bruker APEX CCD area-detector diffractometer
Absorption correction: multi-scan (*SADABS*; Sheldrick, 2008 ▶) *T*_min_ = 0.944, *T*_max_ = 0.994
7386 measured reflections
1516 independent reflections
1124 reflections with *I* > 2σ(*I*)
*R*_int_ = 0.074

#### Refinement

*R*[*F* ^2^ > 2σ(*F* ^2^)] = 0.043
*wR*(*F* ^2^) = 0.114
*S* = 0.95
1516 reflections
127 parameters
H-atom parameters constrained
Δρ_max_ = 0.42 e Å^−3^
Δρ_min_ = −0.27 e Å^−3^

### 

Data collection: *SMART* (Bruker, 2001 ▶); cell refinement: *SAINT* (Bruker, 2001 ▶); data reduction: *SAINT*; program(s) used to solve structure: *SHELXS97* (Sheldrick, 2008 ▶); program(s) used to refine structure: *SHELXL97* (Sheldrick, 2008 ▶); molecular graphics: *SHELXTL* (Sheldrick, 2008 ▶) and *ORTEPIII* (Burnett & Johnson, 1996 ▶); software used to prepare material for publication: *SHELXTL*.

## Supplementary Material

Crystal structure: contains datablock(s) I, global. DOI: 10.1107/S160053681205146X/bg2493sup1.cif

Structure factors: contains datablock(s) I. DOI: 10.1107/S160053681205146X/bg2493Isup2.hkl

Supplementary material file. DOI: 10.1107/S160053681205146X/bg2493Isup3.mol

Supplementary material file. DOI: 10.1107/S160053681205146X/bg2493Isup4.cml

Additional supplementary materials:  crystallographic information; 3D view; checkCIF report

## Figures and Tables

**Table 1:** Hydrogen-bond geometry (Å, °)

*D*—H⋯*A*	*D*—H	H⋯*A*	*D*⋯*A*	*D*—H⋯*A*
N1—H1*A*⋯O1^i^	0.86	2.17	2.982 (3)	159
N1—H1*B*⋯O1^ii^	0.86	2.33	3.097 (4)	149
O2—H21⋯N5^iii^	0.82	1.89	2.708 (2)	176
N5—H51⋯O3^iv^	0.98	1.91	2.783 (3)	147

Symmetry codes: (i) 
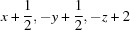
; (ii) 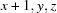; (iii) 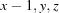; (iv) 
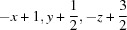
.

**Fig. 1.**
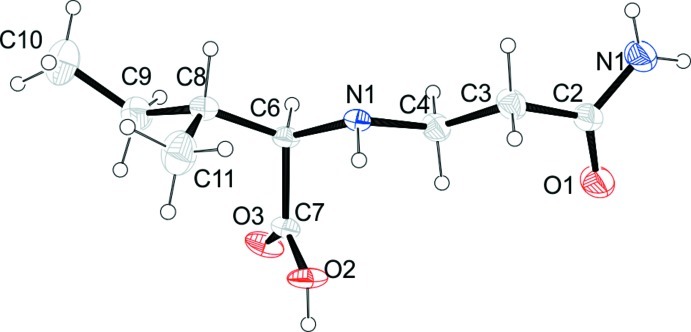
ORTEP representation of the title compound with atomic labeling shown with 30% probability displacement ellipsoids.

**Fig. 2.**
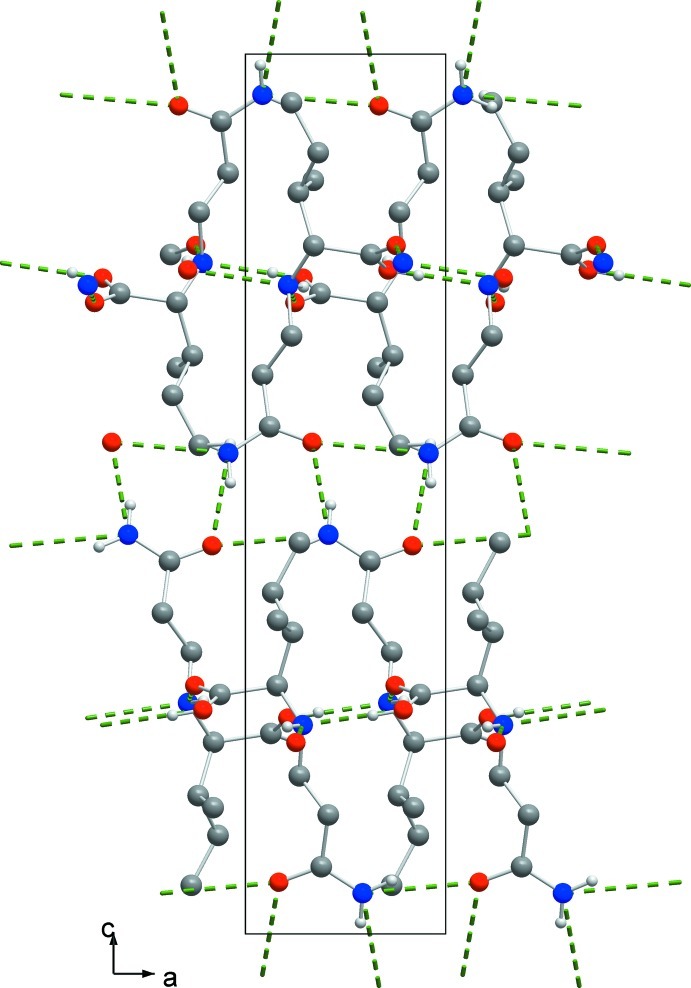
View of the unit cell of the title compound along [010]. Hydrogen bonds are drawn as dashed green lines. For clarity, hydrogen atoms not involved in the hydrogen bonding are omitted.

## supplementary crystallographic information

### Comment

Acrylamide is a water-soluble unsaturated amide, a reactive monomer and an industrial chemical used in many technological applications.

It is also a contaminant in baked and fried starchy food as a result of Maillard reactions involving asparagine and reducing sugars that leads to disseminated human exposure. So people may be exposed to acrylamide in industry as well as in daily life *via* diet and drinking water. Furthermore, it was recently reported a novel method for the determination of acrylamide in particulare-phase outdoor aerosol (Zangrando *et al.*, 2012).

It is known that acrylamide is a neurotoxin and putative human carcinogen. In the last years a lot of different toxicological investigations have been carried out (Besaratinia and Pfeifer, 2007*:* Parzefall, 2008*;* Bowyer *et al.*, 2009;Wang *et al.*, 2010; Mei *et al.*, 2010; Koyama *et al.*, 2011; Lee *et al.*, 2012; Nixon *et al.*, 2012*).* Therefore, acrylamide was included (with a limit value of 0.1µg/*L*) to the numerous substances to be monitored in drinking water according to EU Water Framework Directive (EU 2000). The best method for the determination of acrylamide in water is the liquid chromatography/ tandem mass spectrometry (LC—MS/MS) (Marin *et al.*, 2006; Lucentini *et al.*, 2009; Keramat *et al.*, 2011). In the area of foods GC method with bromination of acrylamide as a derivatization reaction was used (Tareke *et al.*, 2002; Pittet *et al.*, 2004; Castle & Eriksson, 2005; Mizukami *et al.*, 2006; Dias Soares *et al.*, 2009). But also methods such as high-performance thin-layer chromatography (HPTLC) (Alpmann & Morlock, 2008) and a bioassay of dietary acrylamide exposure on the basis of monoclonal antibodies (Preston *et al.*, 2009) were used.

In toxicological investigations it could be proven, that reactions between acrylamide and different amino acids take place *(*Rice, 2005). These reactions and the corresponding adducts can be used also for the analytical determination of acrylamide in drinking water (Perez & Osterman-Golkar, 2003). There the amino acid isoleucine served as a nucleophilic trapping agent. Our group examined the derivatization of acrylamide with isoleucine in the course of the drinking water analysis.

The molecular structure of the reaction product from acrylamide and isoleucine and the atom-labeling scheme is shown in Fig. 1. The absolute configuration has not been determined by anomalous-dispersion effects in diffraction measurements on the crystal, but assigned by reference to an unchanging chiral centre in the synthetic procedure. Each molecule forms six hydrogen bonds to six adjacent molecules leading to a three-dimensional-network structure. In the a-c plane adjacent molecules form strong hydrogen bonds between amino donor groups and oxygen acceptor atoms.Each molecule is further involved in N—H···O bonds parallel the crystallographic b direction. The hydrogen bond network is completed by a further hydrogen bond between a hydroxy donor group and a nitrogen acceptor atom parallel to the a direction. The resulting arrangement together with the hydrogen bonding system (dashed green lines) is shown in Fig. 2.

### Experimental

The derivatization of acrylamide (for synthesis, > 99%; Merck, Darmstadt, Germany) with *L*-isoleucine (Biochemica > 99%; Fluka, Deishofen, Germany) was achieved in a water bath at 39 °C. For the reaction 0.4233 g *L*-isoleucin (3.2 mol) were dissolved in water (19.8 g) and temperated to 30 °C. The pH was set to 10 with sodium hydroxide (2*M)* and 0.4562 g (6.4 mol) acrylamide was added. The flask was shaken for two minutes and placed in the water bath for 48 h. Crystallized solids were filtered out, washed with cold methanol, redissolved in small amounts of hot water and at 4 °C for one week to yield light yellow crystals with a melting point of 282 °C and a purity (DSC) of 99.9%.

### Refinement

All H-atoms were positioned geometrically and refined using a riding model with d(C—H) = 0.93 Å, *U*_iso_=1.2*U*_eq_ (C) for aromatic C atoms, 0.98 Å, *U*_iso_ = 1.2*U*_eq_ (C) for CH, 0.97 Å, *U*_iso_= 1.2*U*_eq_ (C) for CH_2_, 0.96 Å, *U*_iso_ = 1.5*U*_eq_ (C) for CH_3_ hydrogen atoms, and d(N—H) = 0.86 Å, *U*_iso_=1.2*U*_eq_ (N). In the absence of significant anomalous dispersion effects Friedel pairs were merged. The absolute configuration has not been determined by anomalous-dispersion effects in diffraction measurements of the crystal, but assigned as based on an unchanged chiral centre in the synthetic procedure.

### Crystal data

**Table:**

C_9_H_18_N_2_O_3_	*F*(000) = 440
*M**_r_* = 202.25	*D*_x_ = 1.207 Mg m^−^^3^
Orthorhombic, *P*2_1_2_1_2_1_	Mo *K*α radiation, λ = 0.71073 Å
Hall symbol: P 2ac 2ab	Cell parameters from 1516 reflections
*a* = 5.2989 (17) Å	θ = 1.8–27.5°
*b* = 9.024 (3) Å	µ = 0.09 mm^−^^1^
*c* = 23.268 (7) Å	*T* = 298 K
*V* = 1112.6 (6) Å^3^	Needle, colourless
*Z* = 4	0.64 × 0.06 × 0.06 mm

### Data collection

**Table:**

Bruker APEX CCD area-detector diffractometer	1516 independent reflections
Radiation source: fine-focus sealed tube	1124 reflections with *I* > 2σ(*I*)
Graphite monochromator	*R*_int_ = 0.074
ω/2θ scans	θ_max_ = 27.5°, θ_min_ = 1.8°
Absorption correction: multi-scan (*SHELXTL* [*SADABS*?]; Sheldrick, 2008)	*h* = −6→6
*T*_min_ = 0.944, *T*_max_ = 0.994	*k* = −10→11
7386 measured reflections	*l* = −30→25

### Refinement

**Table:**

Refinement on *F*^2^	Primary atom site location: structure-invariant direct methods
Least-squares matrix: full	Secondary atom site location: difference Fourier map
*R*[*F*^2^ > 2σ(*F*^2^)] = 0.043	Hydrogen site location: inferred from neighbouring sites
*wR*(*F*^2^) = 0.114	H-atom parameters constrained
*S* = 0.95	*w* = 1/[σ^2^(*F*_o_^2^) + (0.0694*P*)^2^] where *P* = (*F*_o_^2^ + 2*F*_c_^2^)/3
1516 reflections	(Δ/σ)_max_ < 0.001
127 parameters	Δρ_max_ = 0.42 e Å^−^^3^
0 restraints	Δρ_min_ = −0.27 e Å^−^^3^

### Special details

**Table:**

Geometry. All e.s.d.'s (except the e.s.d. in the dihedral angle between two l.s. planes) are estimated using the full covariance matrix. The cell e.s.d.'s are taken into account individually in the estimation of e.s.d.'s in distances, angles and torsion angles; correlations between e.s.d.'s in cell parameters are only used when they are defined by crystal symmetry. An approximate (isotropic) treatment of cell e.s.d.'s is used for estimating e.s.d.'s involving l.s. planes.
Refinement. Refinement of *F*^2^ against ALL reflections. The weighted *R*-factor *wR* and goodness of fit *S* are based on *F*^2^, conventional *R*-factors *R* are based on *F*, with *F* set to zero for negative *F*^2^. The threshold expression of *F*^2^ > σ(*F*^2^) is used only for calculating *R*-factors(gt) *etc*. and is not relevant to the choice of reflections for refinement. *R*-factors based on *F*^2^ are statistically about twice as large as those based on *F*, and *R*- factors based on ALL data will be even larger.

### Fractional atomic coordinates and isotropic or equivalent isotropic displacement parameters (Å^2^)

**Table:**

	*x*	*y*	*z*	*U*_iso_*/*U*_eq_	
C10	0.7698 (9)	0.2828 (6)	0.55528 (14)	0.1012 (15)	
H102	0.6633	0.2437	0.5256	0.152*	
H101	0.9239	0.2271	0.5568	0.152*	
H103	0.8073	0.3848	0.5472	0.152*	
C9	0.6365 (7)	0.2719 (4)	0.61206 (12)	0.0594 (8)	
H9A	0.6549	0.1718	0.6267	0.071*	
H9B	0.4579	0.2899	0.6062	0.071*	
C11	0.6607 (8)	0.5381 (3)	0.64355 (12)	0.0669 (10)	
H11A	0.7246	0.6032	0.6728	0.100*	
H11B	0.4800	0.5454	0.6422	0.100*	
H11C	0.7297	0.5661	0.6070	0.100*	
C8	0.7355 (5)	0.3811 (3)	0.65718 (9)	0.0416 (6)	
H8	0.9202	0.3770	0.6555	0.050*	
C6	0.6585 (4)	0.3299 (3)	0.71788 (9)	0.0303 (5)	
H61	0.7165	0.2210	0.7210	0.036*	
C7	0.3729 (4)	0.3374 (3)	0.72786 (10)	0.0325 (5)	
O3	0.2475 (3)	0.22453 (18)	0.71760 (8)	0.0472 (5)	
O2	0.2878 (3)	0.45814 (17)	0.74539 (7)	0.0461 (5)	
H21	0.1361	0.4499	0.7516	0.069*	
N5	0.7864 (3)	0.42102 (19)	0.76255 (7)	0.0287 (4)	
H51	0.7098	0.5195	0.7647	0.034*	
C4	0.7693 (5)	0.3514 (3)	0.82032 (9)	0.0387 (6)	
H41	0.8359	0.2514	0.8184	0.046*	
H42	0.5935	0.3452	0.8317	0.046*	
C3	0.9138 (5)	0.4382 (3)	0.86474 (10)	0.0437 (6)	
H31	0.8541	0.5398	0.8653	0.052*	
H32	1.0916	0.4392	0.8549	0.052*	
C2	0.8789 (5)	0.3691 (3)	0.92360 (11)	0.0441 (6)	
O1	0.6669 (4)	0.3386 (3)	0.94118 (8)	0.0640 (7)	
N1	1.0852 (5)	0.3443 (3)	0.95370 (10)	0.0571 (7)	
H1A	1.0748	0.3050	0.9873	0.069*	
H1B	1.2303	0.3674	0.9398	0.069*	

### Atomic displacement parameters (Å^2^)

**Table:**

	*U*^11^	*U*^22^	*U*^33^	*U*^12^	*U*^13^	*U*^23^
C10	0.107 (4)	0.145 (4)	0.051 (2)	0.017 (4)	0.002 (2)	−0.022 (2)
C9	0.0570 (19)	0.073 (2)	0.0485 (16)	0.0103 (17)	−0.0078 (16)	−0.0115 (14)
C11	0.087 (3)	0.0601 (19)	0.0532 (16)	−0.0004 (19)	0.0045 (18)	0.0146 (14)
C8	0.0266 (12)	0.0569 (16)	0.0414 (13)	0.0032 (12)	0.0010 (11)	0.0006 (11)
C6	0.0201 (10)	0.0329 (11)	0.0379 (12)	0.0000 (9)	−0.0006 (9)	−0.0035 (9)
C7	0.0210 (10)	0.0351 (13)	0.0414 (12)	0.0018 (10)	−0.0020 (10)	0.0028 (10)
O3	0.0258 (9)	0.0366 (9)	0.0792 (13)	−0.0046 (8)	−0.0105 (9)	0.0016 (8)
O2	0.0176 (7)	0.0442 (10)	0.0765 (12)	0.0006 (7)	0.0048 (8)	−0.0150 (8)
N5	0.0205 (8)	0.0306 (9)	0.0351 (9)	0.0005 (8)	0.0001 (7)	0.0010 (7)
C4	0.0326 (12)	0.0442 (14)	0.0391 (13)	−0.0061 (12)	−0.0010 (11)	0.0080 (10)
C3	0.0359 (14)	0.0528 (16)	0.0423 (14)	−0.0086 (12)	−0.0040 (11)	0.0059 (12)
C2	0.0343 (13)	0.0568 (17)	0.0412 (13)	−0.0005 (12)	−0.0004 (12)	0.0029 (12)
O1	0.0384 (11)	0.1044 (19)	0.0493 (11)	−0.0072 (12)	0.0022 (9)	0.0191 (11)
N1	0.0412 (13)	0.087 (2)	0.0436 (12)	0.0016 (13)	−0.0024 (11)	0.0155 (12)

### Geometric parameters (Å, º)

**Table:**

C10—C9	1.501 (5)	C7—O3	1.239 (3)
C10—H102	0.9600	C7—O2	1.248 (3)
C10—H101	0.9600	O2—H21	0.8200
C10—H103	0.9600	N5—C4	1.487 (3)
C9—C8	1.532 (4)	N5—H51	0.9781
C9—H9A	0.9700	C4—C3	1.506 (3)
C9—H9B	0.9700	C4—H41	0.9700
C11—C8	1.506 (4)	C4—H42	0.9700
C11—H11A	0.9600	C3—C2	1.516 (3)
C11—H11B	0.9600	C3—H31	0.9700
C11—H11C	0.9600	C3—H32	0.9700
C8—C6	1.541 (3)	C2—O1	1.226 (3)
C8—H8	0.9800	C2—N1	1.318 (3)
C6—N5	1.489 (3)	N1—H1A	0.8600
C6—C7	1.532 (3)	N1—H1B	0.8600
C6—H61	1.0319		
			
C9—C10—H102	109.5	C7—C6—H61	109.0
C9—C10—H101	109.5	C8—C6—H61	105.7
H102—C10—H101	109.5	O3—C7—O2	125.9 (2)
C9—C10—H103	109.5	O3—C7—C6	117.7 (2)
H102—C10—H103	109.5	O2—C7—C6	116.4 (2)
H101—C10—H103	109.5	C7—O2—H21	109.5
C10—C9—C8	113.5 (3)	C4—N5—C6	111.72 (17)
C10—C9—H9A	108.9	C4—N5—H51	108.1
C8—C9—H9A	108.9	C6—N5—H51	110.5
C10—C9—H9B	108.9	N5—C4—C3	111.69 (19)
C8—C9—H9B	108.9	N5—C4—H41	109.3
H9A—C9—H9B	107.7	C3—C4—H41	109.3
C8—C11—H11A	109.5	N5—C4—H42	109.3
C8—C11—H11B	109.5	C3—C4—H42	109.3
H11A—C11—H11B	109.5	H41—C4—H42	107.9
C8—C11—H11C	109.5	C4—C3—C2	110.1 (2)
H11A—C11—H11C	109.5	C4—C3—H31	109.6
H11B—C11—H11C	109.5	C2—C3—H31	109.6
C11—C8—C9	111.8 (2)	C4—C3—H32	109.6
C11—C8—C6	113.9 (2)	C2—C3—H32	109.6
C9—C8—C6	110.2 (2)	H31—C3—H32	108.2
C11—C8—H8	106.9	O1—C2—N1	123.0 (2)
C9—C8—H8	106.9	O1—C2—C3	120.3 (2)
C6—C8—H8	106.9	N1—C2—C3	116.7 (2)
N5—C6—C7	108.63 (18)	C2—N1—H1A	120.0
N5—C6—C8	110.73 (18)	C2—N1—H1B	120.0
C7—C6—C8	112.77 (19)	H1A—N1—H1B	120.0
N5—C6—H61	110.0		
			
C10—C9—C8—C11	71.4 (4)	N5—C6—C7—O2	35.8 (3)
C10—C9—C8—C6	−160.9 (3)	C8—C6—C7—O2	−87.3 (3)
C11—C8—C6—N5	−62.4 (3)	C7—C6—N5—C4	70.3 (2)
C9—C8—C6—N5	171.1 (2)	C8—C6—N5—C4	−165.35 (18)
C11—C8—C6—C7	59.6 (3)	C6—N5—C4—C3	176.1 (2)
C9—C8—C6—C7	−66.9 (3)	N5—C4—C3—C2	176.6 (2)
N5—C6—C7—O3	−144.6 (2)	C4—C3—C2—O1	−49.4 (4)
C8—C6—C7—O3	92.2 (3)	C4—C3—C2—N1	130.4 (3)

### Hydrogen-bond geometry (Å, º)

**Table:**

*D*—H···*A*	*D*—H	H···*A*	*D*···*A*	*D*—H···*A*
N1—H1*A*···O1^i^	0.86	2.17	2.982 (3)	159
N1—H1*B*···O1^ii^	0.86	2.33	3.097 (4)	149
O2—H21···N5^iii^	0.82	1.89	2.708 (2)	176
N5—H51···O3^iv^	0.98	1.91	2.783 (3)	147

Symmetry codes: (i) *x*+1/2, −*y*+1/2, −*z*+2; (ii) *x*+1, *y*, *z*; (iii) *x*−1, *y*, *z*; (iv) −*x*+1, *y*+1/2, −*z*+3/2.
